# *Armillaria altimontana* in North America: Biology and Ecology

**DOI:** 10.3390/jof9090904

**Published:** 2023-09-04

**Authors:** Mee-Sook Kim, John W. Hanna, Geral I. McDonald, Ned B. Klopfenstein

**Affiliations:** 1 Pacific Northwest Research Station, USDA Forest Service, Corvallis, OR 97331, USA; 2 Rocky Mountain Research Station, USDA Forest Service, Moscow, ID 83843, USA; john.w.hanna@usda.gov (J.W.H.);

**Keywords:** *Armillaria altimontana*, biological control, potential distribution, climate change

## Abstract

*Armillaria altimontana* is a fungus (Basidiomycota, Agaricomycetes, Agaricales, and Physalacriaceae) that is generally considered as a weak/opportunistic pathogen or saprophyte on many tree hosts. It widely occurs across the northwestern USA to southern British Columbia, Canada, but relatively little is known about its ecological role in the diverse forest ecosystems where it occurs. This review summarizes the biology and ecology of *A*. *altimontana*, including its identification, life cycle, distribution, host associations, and bioclimatic models under climate change.

## 1. Introduction

Within the Basidiomycota (Agaricomycetes, Agaricales, and Physalacriaceae), *Armillaria* is well known as a cause of Armillaria root disease of diverse woody plants that can result in major growth losses (decreased C sequestration) and mortality of forest/horticultural trees. However, pathogenic *Armillaria* can also provide ecological benefits, such as creating openings for forest regeneration by eliminating maladapted trees, augmenting forest succession, and providing wildlife habitats [1,2,3,4]. *Armillaria* species also display diverse ecological functions, such as a “white-rot” decomposer and recycler of organic matter, potential *in situ* biological control agent against an *Armillaria* pathogen [5], mycorrhizal symbiont of orchids in eastern Asia [6,7,8], and symbiotic host or parasite of other fungi [6]. *Armillaria* species are also known for their bioluminescence [9] and widespread/long-term occupancy of a site [10,11]. The genus *Armillaria* comprises more than 37 currently recognized species that are largely distinct between the Northern Hemisphere and Southern Hemisphere, with distinct groups of species occurring within Asia, Europe, Africa, Australia, North America, and South America [3,4]. Following the assignment of exannulate species to *Desarmillaria* [12], 10 species of *Armillaria* are currently recognized in North America: *A*. *solidipes* (formerly known as North American *A*. *ostoyae*), *A*. *gemina*, *A*. *calvescens*, *A*. *sinapina*, *A*. *mellea*, *A*. *gallica*, *A*. *nabsnona*, *A*. *altimontana*, *A*. *cepistipes*, and *A*. *mexicana*.

*Armillaria altimontana* was first recognized as an unnamed North American Biological Species (NABS) X on the basis of mating tests [13], and it was formally described by Brazee et al. [14]. Although *A*. *altimontana* widely occurs across the northwestern USA to southern British Columbia, Canada, relatively little has been previously reported about its ecological role in the diverse forest ecosystems where it occurs. Perhaps *A*. *altimontana* was previously overlooked as a subject of focused studies because it only produces basidiomata sporadically, and these can be observed in locations that are not immediately associated with a host tree. For this reason, studies of *A*. *altimontana* typically require identification based on its vegetative state (e.g., rhizomorph, mycelial fan, or mycelia within colonized organic matter) that typically requires specialized survey methods, such as root excavation and/or removing bark on the lower stem or major roots to expose mycelial fans, rhizomorphs, and/or colonized wood [15,16]. While *A. altimontana* is occasionally found as a pathogen causing Armillaria root disease, it is often only found in a saprophytic state, such as epiphytic rhizomorphs, on diverse coniferous and broad-leaved trees/shrubs. Recently, analyses by Warwell et al. [5] suggested that *A*. *altimontana* can act as an *in situ* biological control agent for Armillaria root disease by competitively excluding the pathogenic *A*. *solidipes* within the soil of a western white pine (*Pinus monticola*) plantation in northern Idaho, USA. Such preliminary information suggests that *A*. *altimontana* could be playing a major, but often undetected, role in forest ecosystem processes. In this review, we will particularly focus on the biology and ecology of *A*. *altimontana*, including its identification, life cycle, distribution, host associations, and bioclimatic models under projected climate change.

## 2. Identification

**Figure 1:**
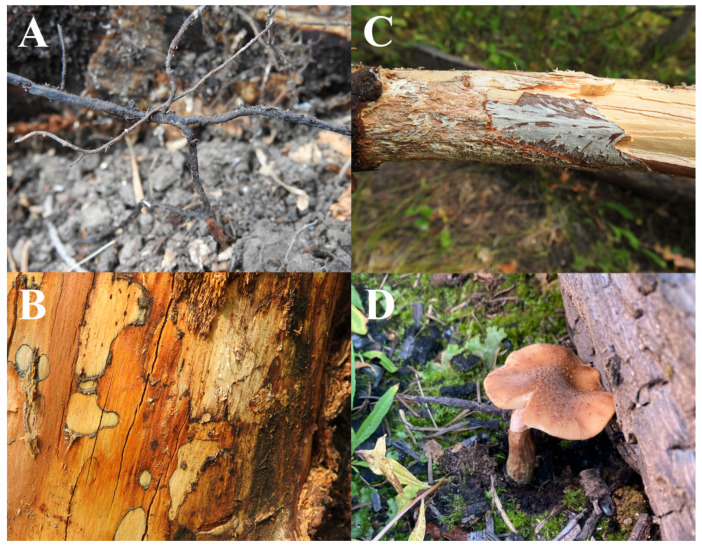
*Armillaria altimontana*. (**A**) Extensive rhizomorph networks are found in association with conifer and hardwood roots and soil/detritus across the forest ecosystem; (**B**) Mycelial fans are observed beneath the bark of dead and dying trees; (**C**) Wood rot with characteristic zone lines; and (**D**) fruiting bodies (basidiomata) are frequently produced in solitary or couplets and are often found on soil/detritus without obvious associations with a host tree.

Methods to identify *A*. *altimontana* have evolved over time, paralleling the methods to identify other *Armillaria* spp.; however, *A*. *altimontana* can be difficult to identify based solely on basidioma morphology, as this species is typically found in its vegetative state (e.g., rhizomorphs or mycelia; Figure 1A,B) and morphological characters of its basidioma (Figure 1D) can vary or overlap with other *Armillaria* spp. [14]. Prior to DNA-based methods, *A*. *altimontana* (as NABS X) was identified through mating tests, which required presumed haploid mycelia derived from basidiospores produced by a basidioma [13]. *In vitro* mating tests revealed that *A*. *altimontana* had a low level of compatibility with *A*. *sinapina* (3.5%) and *A*. *cepistipes* (as NABS XI; 6.5%), which was attributed to an evolutionary status that allowed low-level interfertility *in vitro*, but did not significantly contribute to the genomic structure of the respective species [17]. Subsequent studies used flow cytometry, fluorescence microscopy, and PCR-based techniques to confirm that the *in vitro* mating of *A*. *altimontana* can occur with *A*. *sinapina* and *A*. *cepistipes* [18]. However, interspecific hybridization of *A*. *altimontana* has not been documented in nature.

As *A*. *altimontana* samples are usually derived from somatic tissue (e.g., rhizomorphs or mycelia; Figure 1A–C), which is presumed to be diploid, other methods were developed for identification based on somatic tissue. Vegetative incompatibility tests can be used to identify *Armillaria* species based on the confrontation zone reactions, e.g., pseudosclerotial plates (“black lines”) produced by confrontations between isolates of different species vs. colorless antagonism produced by confrontations between isolates of the same species during *in vitro* pairing of an unknown diploid isolate against diploid isolates of a known species, as outlined previously [19,20]. Alternatively, *Armillaria* identification can be based on *in vitro* pairing reactions of an unknown diploid isolate against a known haploid isolate, as covered in a previous report [21].

The utility of DNA sequences for *Armillaria* was demonstrated in the phylogenetic analyses of the intergenic spacer region 1 (IGS1) between the 3′ end of the 26S and the 5S region of rDNA [22], which subsequently served as the basis for a polymerase chain reaction- (PCR-) and restriction endonuclease-based diagnostic test [23]. Although the IGS1 region is useful in distinguishing *A*. *altimontana*, sequence analyses are hampered by multiple SNPs, and IGS1 is not reliable for distinguishing all North American species of *Armillaria* [24,25]. Subsequently, translation elongation factor-1α (*tef1*) was found to be reliable for separating among 11 known *Armillaria*/*Desarmillaria* species of North America [26,27,28,29], and other loci were also shown to be useful to further resolve the phylogenetic relationships among the armillarioid fungi [12].

## 3. Life Cycle

The life cycle of *A*. *altimontana* is not definitively established, so it is largely inferred from partial information and that of other *Armillaria* [4]. The vegetative state (rhizomorphs and mycelia within the soil and colonized wood/organic matter; Figure 1A–C) of *A*. *altimontana* was determined to be diploid (0.152 pg DNA/nucleus) with a single nucleus per cell [24]; meanwhile, its basidiospore-derived mycelium is believed to initially be in the haploid status, but the nuclear DNA content can double under prolonged culture conditions, resulting in a doubled-haploid nuclear status that behaves as a haploid in mating tests [18].

The basidiomata of *A*. *altimontana* are only apparently produced rarely or sporadically [14]. For example, *A*. *altimontana* commonly occurred across a western white pine planting in northern Idaho; however, its basidiomata were never observed over a 10-year observation period or in occasional plot surveys thereafter [30]. When a basidioma is produced, it may be solitary on the forest floor without any obvious association with a host tree (McDonald, unpublished observations). Of further note is that *A*. *altimontana* basidiomata were previously produced *in vitro* using orange (*Citrus* sp.) fruit slices as a substrate [31]. As with most North American *Armillaria*, *A*. *altimontana* exhibits a bifactorial heterothallism [13,32]. Basidiospores or basidiospore-derived mycelia with compatible mating alleles can mate and likely form transient heterokaryons that are typically maintained through transient clamp connections [33] before karyogamy occurs to create diploid nuclei, which are maintained as one nucleus per cell via mitosis. The diploid mycelia of *Armillaria* are likely capable of surviving for decades in woody debris [34], and individual genets (vegetative clones) of *A*. *altimontana* can apparently exist on a site for at least centuries [5].

## 4. Distribution

**Figure 2:**
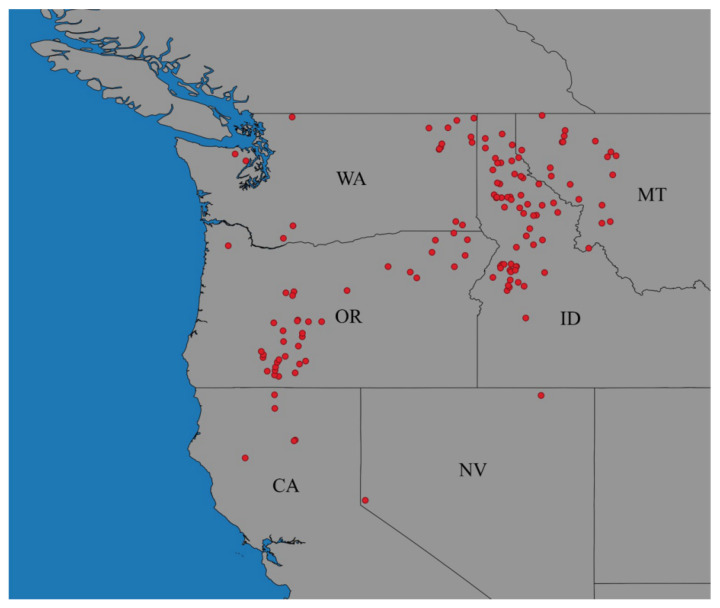
Documented collection points of DNA sequence-confirmed *Armillaria altimontana* across the western states of the USA (California, Idaho, Montana, Nevada, Oregon, and Washington). Note: *Armillaria altimontana* has also been documented in southwestern Canada (British Columbia), but these locations were not obtained for this study. Documented collection points are listed in Table S1.

Surveys of northern Idaho, eastern Washington, western Montana, and northwestern Oregon in the USA documented the common occurrence of *A*. *altimontana* across diverse forest sites in these regions [35,36] (Table S1; Figure 1 and Figure 2), and its occurrence was also documented on diverse sites in the East Cascades of Oregon [37,38] (Table S1).

The known northern distribution of *A*. *altimontana* extends to southeastern British Columbia [39]; whereas, its known southern distribution appears in the Klamath Mountains of the Coast Ranges of California and in the mountains east of Lake Tahoe in Nevada, USA [40] (Table S1). It has been found at an elevation range from 484 m (OR, USA) to 2070 m (NV, USA) (Table S1) [40].

The eastern and southern limits of *A*. *altimontana* can also be inferred from *Armillaria* surveys where it has not been found. Surveys of 180 plots in Wyoming, USA did not reveal any *A*. *altimontana* [41,42], nor did surveys of 16 random plots in Utah, USA forests [35]; however, both surveys revealed the presence of other *Armillaria* spp. In addition, *A*. *altimontana* was not found in USA surveys of Arizona, New Mexico, Colorado, or southwestern South Dakota where *A*. *solidipes* (as *A*. *ostoyae*) was found [41,43,44,45], and it was not found in forest surveys of the Canadian prairie provinces of Alberta, Saskatchewan, and Manitoba where *A*. *solidipes* (as *A*. *ostoyae*), *A*. *sinapina*, and/or *A*. *calvescens* were found [46]. Although it remains possible that *A*. *altimontana* will be found in specific locations within states and provinces where it has not yet been documented, *A*. *altimontana* does not appear to be a major component of forest ecosystems in these regions. It should also be noted that the known distribution of *A*. *altimontana* is likely underrepresented in areas where surveys focused on diseased trees. The range of *A*. *altimontana* appears limited to western North America, and it has not been found in surveys of the Southern Hemisphere and Eurasia, according to previous reports [7,28,47,48,49,50].

## 5. Bioclimatic Models to Determine the Climatic Influences on Potential Distribution

Previously, maximum entropy (MaxEnt) bioclimatic modeling [51] was demonstrated as an effective tool to predict the suitable climate space (potential distribution) of *A*. *solidipes* under contemporary and projected future climate scenarios [16]. The MaxEnt approach to bioclimatic modeling is well suited for *Armillaria* as these algorithms perform well with limited occurrence points [52,53] and presence-only occurrence data [54]. For these reasons, MaxEnt bioclimatic modeling was used to predict the potential distribution of *A*. *altimontana* and the climatic factors that influence its occurrence. A total of 139 global positioning system (GPS) locations for presence of DNA sequence-confirmed *A*. *altimontana* were obtained from previous studies [35,36,37,38,55] and other surveys that are both shown in Table S1 and Figure 2.

**Figure 3:**
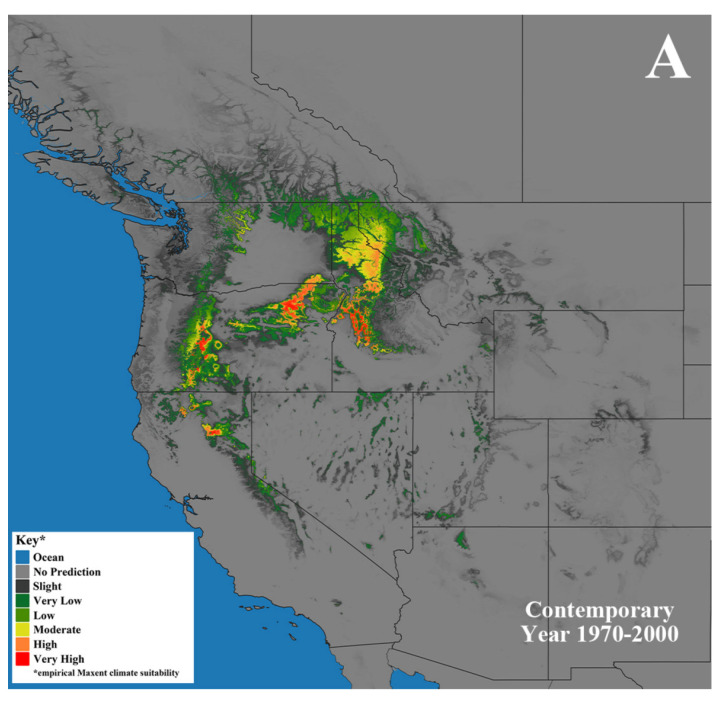

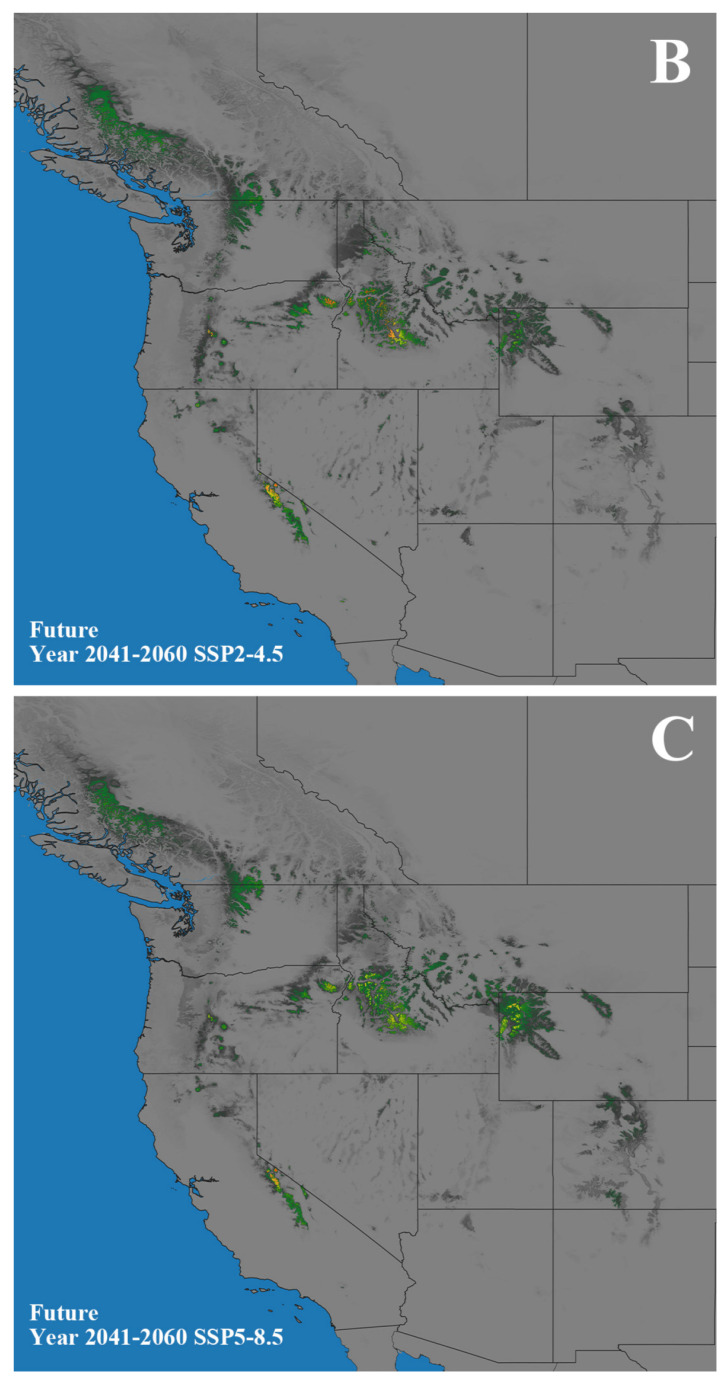

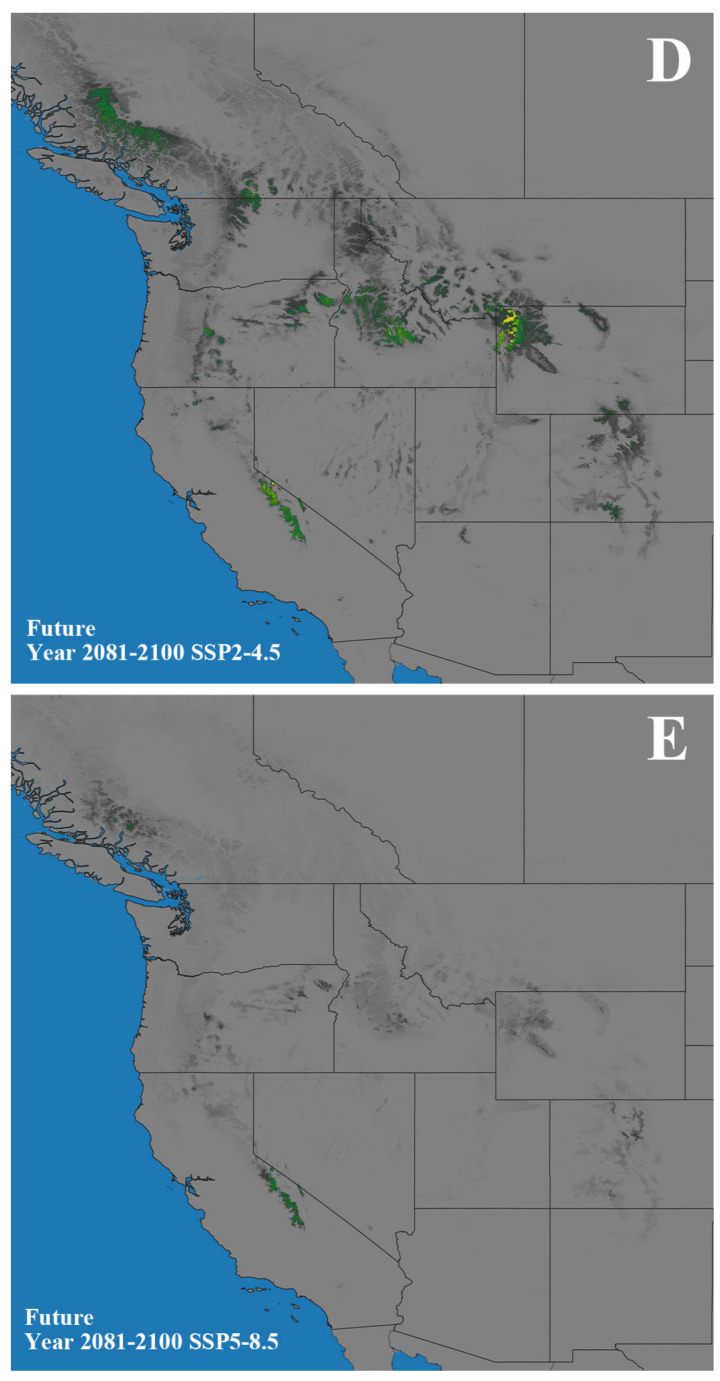
MaxEnt-based bioclimatic models for *Armillaria altimontana* in western North America. (**A**) Contemporary years (1970–2000). Two different shared socioeconomic pathway (SSP) scenarios were used for future bioclimatic models of two different time periods: (**B**) the years 2041–2060 SSP2-4.5, (**C**) the years 2041–2060 SSP5-8.5, (**D**) the years 2081–2100 SSP2-4.5, and (**E**) the years 2081–2100 SSP5-8.5. Darkest gray represents the predicted empirical MaxEnt climate suitability, with dark green, light green, yellow, orange, and red indicating increased probabilities of climatic suitability, respectively.

The MaxEnt bioclimatic modeling and evaluation methods largely followed the protocol of Kim et al. [16], in which Worldclim version 2.1 [56] provided 19 bioclimatic variables (Table 1) under a 30 s (ca. 1 km^2^) spatial resolution that were used in the species distribution model for *A*. *altimontana* under the contemporary (1970–2000) time period (Figure 3A). For this predictive model, mean temperature of the wettest quarter (Bio8), precipitation of the driest quarter (Bio17), and precipitation seasonality (Bio15) were the climate variables that exhibited the highest permutation importance (Table 1). This model was computed from 20 replicate runs using cross validation, which scored an average AUC score of 0.978 with a standard deviation of 0.010. An AUC score of one represents a perfect model, while values below 0.5 are worse than random [57].

Predictions from the MaxEnt bioclimatic models (Figure 3A) largely concurred with the *A*. *altimontana* occurrence data found from the surveys. These predictions demonstrated a high probability of suitable climate space for *A. altimontana* in northwestern Montana, northern Idaho, northeastern Washington, northeastern Oregon, the Oregon Cascade Mountains in the USA, and southeastern British Columbia, Canada. Interestingly, the projected range of *A*. *altimontana* is congruent with the range of the western white pine in many areas. Based on the overlapping ranges and the mutualistic ecology of *A*. *altimontana* and western white pine, we hypothesize that these two ecosystem components have a long co-evolutionary history; however, more in depth studies are needed to examine this hypothesis.

Predictive distribution models based on future climate scenarios were also projected for two future time periods (for the years 2041–2060 and the years 2081–2100) using 30 s (ca. 1 km^2^) resolution CMIP6 downscaled data (WorldClim v 2.1). Two different shared socioeconomic pathways (SSPs) were paired with the “Canadian Ocean Ecosystem model” (CanESM5-CanOE) GCM [58]. In general, five SSP narratives describe the broad socioeconomic trends that could shape future society and we chose to use two scenarios: SSP2-4.5 (“middle of the road” future world where trends do not shift markedly from historical patterns) (Figure 3B–C) and SSP5-8.5 (“fossil-fueled development,” which follows the path of rapid and unconstrained growth in economic output and fossil fuel-based energy use) (Figure 3D–E). The results of the future models indicate that *A. altimontana* may have a substantially reduced range in the future if the predicted models of the future climate come to fruition (Figure 3C–F). It is noteworthy that this trend also parallels the predictions of many host tree species [16,59,60]. Such information provides insights into geographic regions where *A. altimontana* is predicted to potentially occur under contemporary and future climate scenarios. Such results indicate that climate mitigation measures must clearly be a top priority if the goal is to sustain future healthy forest ecosystems that have any resemblance to those of the present and recent past.

## 6. Host Associations

Within the Pacific Northwest of North America, *A*. *altimontana* has been found in association with diverse conifers and hardwoods. In northern California, it was found in association with dead red fir (*Abies magnifica*) in a natural forest, and it was also found in association with disease on a living, planted ponderosa pine (*Pinus ponderosa*) [61]. In comparison, *A*. *altimontana* (as NABS X) was associated with dead grand fir (*Abies grandis*) and a diseased, living Douglas-fir (*Pseudotsuga menziesii*) in northeastern Oregon [11,62]. Surveys in southeastern British Columbia, Canada, revealed *A*. *altimontana* in association with dead conifers, a conifer stump, and a dead birch (*Betula* sp.) [39]. Based on extensive surveys of random plots in Montana, Idaho, Washington, and Oregon, and associated surveys, *A*. *altimontana* was found in association with diverse conifers (e.g., *Abies amabilis*, *A. concolor, A*. *grandis*, *A*. *lasiocarpa*, *A. magnifica*, *Larix occidentalis*, *Picea engelmannii*, *Pinus albicaulis*, *P. contorta*, *P. jeffreyi*, *P. lambertiana*, *P. monticola*, *P. ponderosa*, *Pseudotsuga menziesii, Taxus brevifolia*, *Thuja plicata*, *Tsuga heterophylla*, and *Tsuga mertensiana*), hardwoods (e.g., *Acer*, *Alnus*, *Betula*, *Castanopsis*, *Philadelphus*, *Populus*, *Prunus*, *Salix*, *Sambucus*, and *Sorbus*), shrubs (e.g., *Acer*, *Amelanchier*, *Arctostaphylos*, *Ceanothus*, *Cornus*, *Holodiscus*, *Lonicera*, *Menziesia*, *Paxistima*, *Philadelphus, Purshia*, *Ribes, Rosa*, *Shepherdia*, and *Vaccinium*), and other plants (e.g., *Xerophyllum*) (Table S1; Table 2). In most cases, *A*. *altimontana* was collected as epiphytic rhizomorphs on the outside of roots and in the rhizosphere, with other samples collected from rotten wood, and mycelial fans under the bark of dead/dying trees; however, pathogenic signs of *A*. *altimontana* are infrequently observed as mycelial fans and/or rotten wood on living conifers (e.g., *A*. *grandis* and *L*. *occidentalis*) and hardwoods (e.g., *Alnus*).

## 7. Ecology

The pathogenicity of *A*. *altimontana* is difficult to determine with certainty. As inoculation tests with *Armillaria* are impractical for the most part or unrepresentative of natural conditions, interpretations of pathogenicity must be inferred from symptoms and signs in the field. Indications of pathogenicity are mycelial fans on live trees, mycelial fans on dead trees that exhibit a previous host response to the infection (e.g., resinosis or callousing), or wood rot with mycelia and zone lines on a live tree. The capacity of *A*. *altimontana* to degrade wood also suggests that it can cause wood rot on living trees if it is provided a mode of ingress (e.g., through wounds or insect boring) into the heartwood. Mycelial fans under the bark, rhizomorphs, and/or wood rot on dead trees are not definitive signs/symptoms of disease, and *A*. *altimontana* is frequently found in association with trees that are dead or dying from other causes. Furthermore, *A*. *altimontana* and pathogenic *A*. *solidipes* are occasionally found on the same tree, where *A*. *solidipes* is causing disease. Thus, *A*. *altimontana* is not always definitively acting as a pathogen, even when it is found on a diseased tree. For these reasons, caution is warranted when interpreting the records of *A*. *altimontana* pathogenicity, and it is also essential that the pathogen or fungal associate is identified correctly. Based on the literature [5,14,37], it seems that *A*. *altimontana* can act as a weak pathogen in a few (ca. 6%) situations where it is found; however, it seems likely that the ecological behavior of *A*. *altimontana* varies with the host, geographic region, and environment where it is found.

Both pathogenic *A*. *solidipes* and *A*. *altimontana* commonly co-occur on cool and mesic to warm and wet sites in the inland, northwestern USA, as previously reported [30,36,37]; however, *A*. *solidipes* has not been found in northern California where *A*. *altimontana* was found [61]. In northeastern Oregon, *A. altimontana* (as NABS X) was found in association with stumps of true fir with potential co-occurrence with *Heterobasidion* [62]. Based on a series of random plots in the inland, northwestern USA, McDonald et al. [55] determined that vegetative subseries (groups of habitat types), which reflect temperature and moisture regimes, could be used as a predictor for the occurrence of *A*. *altimontana* (as NABS X). Based on *Armillaria* surveys in the East Cascades of Oregon, *A*. *altimontana*, which comprised some isolates with heterogeneous IGS1 sequences, was collected from the greatest range of climates, as inferred from the vegetation subseries [38]. Although most vegetation subseries support both *A*. *altimontana* and the pathogenic *A*. *solidipes*, a number of warmer and/or drier vegetation subseries only supported *A*. *solidipes*. For example, several sites with their habitat types falling within the Douglas-fir climax of the dry forb group are expected to only be able to support one *Armillaria* species, *A*. *solidipes*, which suggests that these sites are too dry to support *A*. *altimontana* [63].

At diverse forest sites in northern Idaho and eastern Washington, nonpathogenic *A*. *altimontana* (as NABS X) was found on sites that also contained pathogenic *A*. *solidipes* (as *A*. *ostoyae*), and *A*. *solidipes* appeared less frequently in spaces occupied by *A*. *altimontana* [36]. In undisturbed plots in northeastern Washington, *A*. *altimontana* was common on sites that also contained *A*. *solidipes*; however, *A*. *solidipes* was uncommon in spaces dominated by *A*. *altimontana*, which indicated that *A*. *altimontana* may potentially act to competitively exclude pathogenic *A*. *solidipes* [15]. Furthermore, disturbances, such as thinning and fertilization, were associated with a reduced occurrence of *A*. *altimontana*, depending on the vegetation subseries [15]. In areas where *A*. *altimontana* and *A*. *solidipes* co-occur, a low level of Armillaria root disease was found in natural stands; in contrast, Armillaria root disease was more common in stands that were planted, thinned, or near roads [64].

Rhizomorphs are the primary means of vegetative spread by *A*. *altimontana*, which can result in large genets that occupy areas >1 ha. Within its range, rhizomorphs of *A*. *altimontana* are commonly found in association with tree roots, detritus, and the soil, where they have been found at depths of up to 590 mm (McDonald, unpublished observations). Individual genets of *A*. *altimontana* rarely overlap spatially; however, genets of *A*. *altimontana* occasionally overlap genets of *A*. *solidipes* [5,30]. The genet size of *A*. *altimontana* (as NABS X) was ca. 2 ha in a mixed conifer forest in northeastern Oregon [11], which would translate into ca. 360 years in age, using a spread rate of 0.22 m yr^−1^ [65] (2 ha = radius of 79.8 m/0.22 m per yr = 362 yr). Based on a spread rate of 0.22 m yr^−1^ [65], an *A*. *altimontana* genet was estimated to have co-occupied a northern Idaho, USA site with an *A*. *solidipes* genet for at least 248 yr, and likely for much longer [5]. Interestingly, both *Armillaria* spp. displayed disjunctive and patchy patterns of distribution, indicating a waxing and waning of areas occupied by each *Armillaria* species over the past few centuries. As this pattern predates the disturbances of recent anthropogenic causes, it has been assumed that natural events, such a fire, stand succession, insect attack, disease, weather, etc., were the driving forces that influenced the distribution of *A*. *altimontana* and *A*. *solidipes*, but targeted studies are needed to determine the primary drivers of *A*. *altimontana* distribution and ecological behavior.

## 8. Considerations for *A*. *altimontana* in the Biological Control of Armillaria Root Disease

Previous work has demonstrated the potential of *A*. *altimontana* to function as an *in situ* biological control agent against Armillaria root disease caused by *A*. *solidipes* [5]. However, many questions remain about how to best encourage the biological control activities of *A*. *altimontana*. For the purposes of this discussion, it seems prudent to rule out the widespread applications of *A*. *altimontana* inoculum as it is impractical and ignores the ecological requirements of *A*. *altimontana*. Instead, such activities can focus on areas where *A*. *altimontana* (and associated beneficial microbes) and the pathogen (*A*. *solidipes*) already occur. First, surveys are needed to determine the geographic distribution and climatic/environmental requirements of the putative biocontrol agent (*A*. *altimontana*) and the Armillaria root disease pathogen (*A*. *solidipes*), with a focus on identifying the sites where the putative biocontrol agent and pathogen co-occur. Second, the ecological conditions (e.g., climate, soil properties, stand history, stand composition, stand structure, fire history, etc.) where *A*. *altimontana* functions as a biological control agent must be better characterized. Third, the influence of the interacting microbes and other ecosystem components must be better understood. With this information, management techniques can be developed to encourage *A*. *altimontana* and other beneficial microbes in sites where it co-occurs with the pathogenic *A*. *solidipes*. Examples of management methods that can be evaluated for increasing the biological control of Armillaria root disease include minimizing disturbance, increasing organic matter, adjusting the soil pH, including more vegetation that serves as a host for *A*. *altimontana* but not *A*. *solidipes* (e.g., hardwoods and shrubs), and/or using prescribed burning or other management activities that could favor *A*. *altimontana*. Although more studies are needed to unleash the full potential of the biological control of Armillaria root disease by *A*. *altimontana* and its associated microbes, the benefit of such studies are warranted due to the massive mortality and growth loss associated with Armillaria root disease, which can also decrease C sequestration and further exacerbate climate change.

### 8.1. Interactions with the Soil Microbial Community

Almost any ecological role of *Armillaria* is likely to be dependent on the complex interactions with other diverse soil microbes and their associated biotic/abiotic environment. The soil microbial communities associated with *A*. *altimontana* and the pathogenic *A*. *solidipes* were compared on a 45-year-old plantation of western white pine in northern Idaho, USA [66]. In general, no significant differences were observed in the richness and diversity of the bacterial and fungal communities associated with the two *Armillaria* species, although several differences in the composition of the microbial taxa were observed. Several families of fungi (e.g., Atheliaceae, Rhizopogonaceae, and Suillaceae), which contain ectomycorrhizal fungi that could increase plant productivity, were more abundant in association with *A*. *altimontana* [66]. More investigations are needed to determine if and how the interactive roles of *A*. *altimontana* and the soil microbial community contribute to the increased growth of the *A*. *altimontana*-colonized western white pine and the apparent competitive exclusion of the pathogenic *A*. *solidipes* [5,66].

### 8.2. Phylogenetic Relationships

Phylogenetic analyses of *Armillaria* have placed *A. altimontana* in the Gallica superclade/lineage, which is distinct from the Solidipes/ostoyae, Mellea, Mexicana, and Socialis/tabescens (exannulate) superclades/lineages within the Northern Hemisphere [27,28]. In addition to *A. altimontana*, the Gallica superclade contains several clades of *A. gallica* (from Eurasia and North America), several Chinese biological species, several undescribed lineages from China that include pathogens/symbionts of a traditional medicinal herb (*Gastrodia elata*), unnamed *Armillaria* biological species Nag. E (Japan), *A. calvescens* (North America), *A. cepistipes* (North America), and *A. nabsnona* (from North America and Japan) [7,27,28]. The exact phylogenetic relationship of species within the Gallica superclade/lineage differs among analyses based on their different gene regions [28]; however, this superclade/lineage contains several species/lineages that are associated with weak virulence or symbioses [7].

### 8.3. Genomics

Genome sizes of *Armillaria* spp. vary considerably, and the nuclear DNA content of *A*. *altimontana* was determined to be 152 pg, which was intermediate in size among the North American species, excluding *A*. *mexicana*, that were previously measured [24]. Subsequent genomic sequencing with PacBio revealed a 73,739,702 bp genome, which was estimated to be 95.1–96.7% complete, for an *A*. *altimontana* isolate from northern Idaho [66]. Genomic comparisons of *A*. *altimontana* with the co-occurring, pathogenic *A*. *solidipes* revealed a high level of synteny, which suggests that the gene sets are similar between these two *Armillaria* species, despite their contrasting ecological behavior [66]. However, many small differences that were noted between the genomes of *A*. *altimontana* and *A*. *solidipes* provide potential insights into the saprophytic and protective role of *A*. *altimontana*. Furthermore, putative secreted proteins of *A*. *altimontana* provide information about its potential interactions with their hosts and enzymes involved in saprophytic decomposition; meanwhile, enzymes involved in the synthesis of secondary metabolites may influence the associated microbial community [66].

## 9. General Considerations

Although *A*. *altimontana* was first recognized as the NABS X in 1979 [13], and was formally described as a species in 2012 [14], much remains unknown about the ecological behavior of this species in relation to its geographic distribution (e.g., latitude, longitude, elevation, slope, aspect, etc.), host associations, abiotic environment (e.g., temperature, moisture, and soil physical properties), biotic environment (e.g., forest/plant communities, microbial communities, and other biota), and site history (e.g., ecological succession, fire history, silvicultural management, and natural and anthropogenic disturbances). Ecological studies are especially limited by the arduous, time-consuming efforts required to conduct surveys for *A*. *altimontana*, which typically requires the excavation of the tree/woody plant roots and the surrounding soil, recording the geolocation (e.g., GPS) and associated environmental data (e.g., host species, age, size, health status, etc.), collecting vegetative mycelia (e.g., rhizomorphs, mycelial fans, or colonized wood), isolating/culturing the *Armillaria* sample, extracting DNA, conducting PCR, performing DNA sequencing, and identifying the *Armillaria* [16]. DNA sequence-based methods have dramatically improved the speed and efficiency of the *A*. *altimontana* identification process, compared to culture-based pairing tests; however, surveys for *A*. *altimontana* that require root excavation, sample collection, and culture establishment remain laborious. The efficiency of these *Armillaria* surveys will likely be improved through the continued development of metagenomic or metabarcoding methods to detect/identify these *Armillaria* species and associated microbial species based on the environmental DNA (eDNA) within the soil or root samples [66,67,68].

The complex ecological conditions that contribute to *A*. *altimontana* functioning as a forest pathogen, beneficial decomposer, or potential biological control agent that can exclude an Armillaria root disease pathogen, such as *A*. *solidipes*, remain an enigma. The continued development of genomic, metagenomic, transcriptomic, and metatranscriptomic tools for *Armillaria*-associated studies [66,67,69,70,71] will provide avenues to decipher the ecological functioning of *A*. *altimontana* in relation to the complex interactions among the biotic and abiotic environments. One long-term goal of studies that are focused on understanding the ecological interactions of *A*. *altimontana* is to develop novel, ecologically friendly, and sustainable approaches for forest management to reduce Armillaria root disease and increasing forest productivity (C sequestration) and resilience. Such studies can help determine which management activities (e.g., prescribed fire, organic matter retention, soil pH adjustments, favoring/planting appropriate tree species, thinning, etc.) will promote beneficial functions of *A*. *altimontana* and its associated microbes, while decreasing the detrimental activities of *A*. *solidipes* or other forest root pathogens and preparing for climate change.

## Figures and Tables

**Table 1:** Summary of MaxEnt’s permutation importance values for each of the 19 contemporary, bioclimatic variables (Bio1–Bio19) derived from the WorldClim (worldclim.org) database (1970–2000) used to model a suitable climate space (potential distribution) based on the occurrence points of *Armillaria altimontana*.

Variable (units)		Permutation Importance
Bio1	Annual mean temperature (°C)	0.2
Bio2	Mean diurnal range [Mean of monthly (max temp–min temp)] (°C)	1.3
Bio3	Isothermality (Bio2/Bio7) (×100)	2.6
Bio4	Temperature seasonality (SD × 100, Coefficient of variation) (°C)	2.9
Bio5	Maximum temperature of warmest month (°C)	4.2
Bio6	Minimum temperature of coldest month (°C)	4.0
Bio7	Temperature annual range (Bio5–Bio6) (°C)	6.8
**Bio8**	Mean temperature of wettest quarter (°C)	**28.6**
Bio9	Mean temperature of driest quarter (°C)	3.1
Bio10	Mean temperature of warmest quarter (°C)	2.0
Bio11	Mean temperature of coldest quarter (°C)	4.0
Bio12	Annual precipitation (mm)	0.9
Bio13	Precipitation of wettest month (mm)	3.8
Bio14	Precipitation of driest month (mm)	2.1
**Bio15**	Precipitation seasonality (Coefficient of variation) (-)	**8.6**
Bio16	Precipitation of wettest quarter (mm)	0.2
**Bio17**	Precipitation of driest quarter (mm)	**16.5**
Bio18	Precipitation of warmest quarter (mm)	3.8
Bio19	Precipitation of coldest quarter (mm)	4.6

Variables with the greatest permutation importance (>8%) are in bold.

**Table 2:** *Armillaria altimontana* host associations (including epiphytic rhizomorphs).

Plant Symbol *	Scientific Name	Common Name
Coniferous trees		
ABAM	*Abies amabilis*	Pacific silver fir
ABCO	*Abies concolor*	White fir
ABGR	*Abies grandis*	Grand fir
ABLA	*Abies lasiocarpa*	Subalpine fir
ABMA	*Abies magnifica*	Red fir
LAOC	*Larix occidentalis*	Western larch
PIEN	*Picea engelmannii*	Engelmann spruce
PIAL	*Pinus albicaulis*	Whitebark pine
PICO	*Pinus contorta*	Lodgepole pine
PIJE	*Pinus jeffreyi*	Jeffrey pine
PILA	*Pinus lambertiana*	Sugar pine
PIMO	*Pinus monticola*	Western white pine
PIPO	*Pinus ponderosa*	Ponderosa pine
PSME	*Pseudotsuga menziesii*	Douglas fir
TABR	*Taxus brevifolia*	Pacific yew or western yew
THPL	*Thuja plicata*	Western red cedar
TSHE	*Tsuga heterophylla*	Western hemlock
TSME	*Tsuga mertensiana*	Mountain hemlock
Broad-leaved trees		
ACER	*Acer*	Maple
ACCI	*Acer circinatum*	Vine maple
ACGL	*Acer glabrum*	Rocky Mountain maple (shrub or tree)
ALNUS	*Alnus*	Alder
BEPA	*Betula papyrifera*	Paper birch
CACH	*Castanopsis chrysophylla*	Golden chinquapin (shrub or tree)
POTR	*Populus trichocarpa*	Black cottonwood
POTR1	*Polpulus tremuloides*	Quaking aspen
PREM	*Prunus emarginata*	Bitter cherry (shrub or small tree)
PRVI	*Prunus virginiana*	Chokecherry (shrub or small tree)
SASC	*Salix scouleriana*	Scouler’s willow (shrub or small tree)
SALIX	*Salix*	Willow
SAMBU	*Sambucus*	Elderberry (shrub or small tree)
SORBU	*Sorbus*	Mountain ash
Shrubs		
ACCI	*Acer circinatum*	Vine maple
AMAL	*Amelanchier alnifolia*	Western serviceberry (shrub)
ARPA	*Arctostaphylos patula*	Greenleaf *manzanita*
CEVE	*Ceanothus velutinus*	Snowbrush
COSES	*Cornus stolonifera/sericea*	Redosier dogwood (shrub)
HODI	*Holodiscus discolor*	Oceanspray
LOUT2	*Lonicera utahensis*	Utah honeysuckle or red twin berry
MEFE	*Menziesia ferruginea*	Rusty menzesia or false huckleberry
PAMY	*Paxistima myrsinites*	Oregon boxleaf
PHLE	*Philadelphus lewisii*	Lewis’ mock-orange, mockorange
PUTR2	*Purshia tridentata*	Bitterbrush
RICE	*Ribes cereum*	Wax current
ROGY	*Rosa gymnocarpa*	Dwarf rose or wood rose
SHCA	*Shepherdia canadensus*	Canada buffaloberry
VAGL	*Vaccinium globulare/membranaceum*	Common huckleberry
Other plants		
XETE	*Xerophyllum tenax*	Common bear grass

* USDA Plants Database (https://plants.usda.gov). Information obtained from [11,39,61,62] and Table S1.

## Data Availability

Not applicable.

## Supplementary Materials

The following supporting information can be downloaded at: https://www.mdpi.com/article/10.3390/jof9090904/s1, Table S1: *Armillaria altimontana* isolates from the western United States with location, host, and collector information.
