# Supplementary material for: Armillaria altimontana in North America: Biology and Ecology
Source: J Fungi (Basel). 2023 Sep 4;9(9):904. doi: 10.3390/jof9090904 (PMC10532946; doi:10.3390/jof9090904)
Supplement: Supplementary file 1 [file jof-09-00904-s001.zip › jof-2592892-supplementary.pdf]

Supplemental Table S1.

Armillaria altimontana isolates from the western United States with location, host, and collector information.

Isolate ID	Latitude	Longitude	State	Host(s)/Association(s)	Collectors
Deerlodge - 8a	46.24	-113.64	Montana	*Alnus* sp.	Geral McDonald et al.
				Pinus contorta	
Couer d'Alene - 1	47.79	-116.15	Idaho	*Abies grandis*	Geral McDonald et al.
				Acer glabrum	
				Tsuga heterophylla	
				Vaccinium globulare	
				soil	
Couer d'Alene - 2	47.74145	-116.42654	Idaho	*Abies grandis*	Geral McDonald et al.
				Holodiscus discolor	
				Tsuga heterophylla	
				soil	
Couer d'Alene Ð 3	47.87226	-115.97509	Idaho	*Abies grandis*	Geral McDonald et al.
				Acer glabrum	
				Pseudotsuga menziesii	
				Salix sp.	
				soil	
Couer d'Alene - 5	47.45455	-116.07646	Idaho	*Alnus* sp.	Geral McDonald et al.
				Abies grandis	
				Acer glabrum	
				Holodiscus discolor	
				Larix occidentalis	
				Pinus monticola	
				Pseudotsuga menziesii	
				Salix sp.	
				soil	
Couer d'Alene - 7a	47.55967	-116.63242	Idaho	*Abies grandis*	Geral McDonald et al.
				Thuja plicata	
				Tsuga heterophylla	
Couer d'Alene - 8	47.85773	-116.56739	Idaho	*Acer glabrum*	Geral McDonald et al.
				Holodiscus discolor	
				Pseudotsuga menziesii	
Boise - 2b	44.59208	-115.83505	Idaho	*Abies grandis*	Geral McDonald et al.
				Abies lasiocarpa	
				Alnus sp.	
				Larix occidentalis	
				Picea engelmannii	
				Salix sp.	
Payette - 1b	44.48	-116.27	Idaho	*Pinus ponderosa*	Geral McDonald et al.
				Rosa gymnocarpa	
Payette - 4a	44.93891	-115.31355	Idaho	*Cornus stolonifera*	Geral McDonald et al.
				Vaccinium globulare	
Payette - 7a	44.81456	-116.63115	Idaho	*Amelanchier alnifolia*	Geral McDonald et al.
				Pinus ponderosa	
				Prunus virginiana	

				Pseudotsuga menziesii	
Nez Perce - 2	45.87328	-115.78073	Idaho	*Abies grandis*	Geral McDonald et al.
				Amelanchier alnifolia	
				Pinus ponderosa	
				soil	
Nez Perce - 3	46.0602	-115.69107	Idaho	*Abies grandis*	Geral McDonald et al.
				Acer glabrum	
				Amelanchier alnifolia	
				Taxus brevifolia	
				Thuja plicata	
				soil	
Nez Perce - 4	45.87603	-115.77765	Idaho	*Abies grandis*	Geral McDonald et al.
				Acer glabrum	
				Amelanchier alnifolia	
				Pseudotsuga menziesii	
				Xerophyllum tenax	
Nez Perce - 5	46.4033	-115.51975	Idaho	*Abies grandis*	Geral McDonald et al.
				Acer glabrum	
				Betula papyrifera	
				Philadelphus lewisii	
				Pseudotsuga menziesii	
				Thuja plicata	
Nez Perce - 6	45.58564	-116.03269	Idaho	*Abies grandis*	Geral McDonald et al.
				Larix occidentalis	
				Picea engelmannii	
				Pinus contorta	
				Pseudotsuga menziesii	
				Xerophyllum tenax	
				soil	
Nez Perce - 7	45.6495	-115.59648	Idaho	*Abies lasiocarpa*	Geral McDonald et al.
				Picea engelmannii	
				soil	
Nez Perce - 8	45.77058	-115.36798	Idaho	*Abies grandis*	Geral McDonald et al.
				Abies lasiocarpa	
				Acer glabrum	
Kaniksu - 5	48.1985	-116.13812	Idaho	*Alnus* sp.	Geral McDonald et al.
				Pseudotsuga menziesii	
				Sorbus sp.	
				soil	
Kaniksu - 6	48.47576	-116.39545	Idaho	*Holodiscus discolor*	Geral McDonald et al.
St.Joe - 1	47.35566	-115.8652	Idaho	*Pinus contorta*	Geral McDonald et al.
				Pseudotsuga menziesii	
St.Joe - 3	46.86869	-116.18814	Idaho	*Prunus emarginata*	Geral McDonald et al.
				Pseudotsuga menziesii	
				Thuja plicata	
St.Joe - 4	47.39651	-115.91712	Idaho	*Amelanchier alnifolia*	Geral McDonald et al.
				Ceanothus velutinus	

				Prunus emarginata	
St.Joe - 5	46.92623	-116.59932	Idaho	*Abies grandis*	Geral McDonald et al.
				Acer sp.	
				Pinus monticola	
				Pseudotsuga menziesii	
				Thuja plicata	
St.Joe - 7	47.19933	-115.46023	Idaho	*Abies grandis*	Geral McDonald et al.
				Abies lasiocarpa	
				Alnus sp.	
				Pseudotsuga menziesii	
				soil	
Colville - 1	48.63559	-117.77462	Washington	*Amelanchier alnifolia*	Geral McDonald et al.
Colville - 4	48.88078	-117.11522	Washington	*Acer glabrum*	Geral McDonald et al.
				Pinus contorta	
Colville - 7	48.62882	-118.26113	Washington	*Alnus* sp.	Geral McDonald et al.
				Larix occidentalis	
				Pinus contorta	
				Pseudotsuga menziesii	
				Shepherdia canadensus	
				soil	
Colville - 8	48.82262	-117.54915	Washington	*Abies grandis*	Geral McDonald et al.
				Acer glabrum	
				Alnus sp.	
				Amelanchier alnifolia	
				Larix occidentalis	
				Thuja plicata	
				soil	
Umitilla - 1a	44.95121	-118.73723	Oregon	*Abies grandis*	Geral McDonald et al.
				Larix occidentalis	
				Pseudotsuga menziesii	
Umitilla - 2	46.1551	-117.40567	Washington	*Abies grandis*	Geral McDonald et al.
Umitilla - 3	44.80322	-118.57125	Oregon	*Abies grandis*	Geral McDonald et al.
Umitilla - 4	45.0929	-119.30239	Oregon	*Larix occidentalis*	Geral McDonald et al.
				Pinus contorta	
				Pinus ponderosa	
Umitilla - 6	45.94841	-117.62629	Oregon	*Pinus ponderosa*	Geral McDonald et al.
				hardwood	
				soil	
Umitilla - 7	45.76653	-118.09682	Oregon	*Abies lasiocarpa*	Geral McDonald et al.
				Picea engelmannii	
Wallowa Whitman - 2	45.08919	-117.61189	Oregon	*Larix occidentalis*	Geral McDonald et al.
				Alnus sp.	
				Pseudotsuga menziesii	
				soil	
Wallowa Whitman - 3	45.45667	-118.18261	Oregon	*Abies grandis*	Geral McDonald et al.
				Acer glabrum	
				Larix occidentalis	

				Picea engelmannii	
Wallowa Whitman - 4a	45.7772	-117.28027	Oregon	*Abies grandis*	Geral McDonald et al.
Wallowa Whitman - 5	45.37662	-117.33785	Oregon	*Alnus* sp.	Geral McDonald et al.
				Picea engelmannii	
				Salix sp.	
Lolo - 5	46.80952	-114.44147	Montana	*Salix* sp.	Geral McDonald et al.
				Amelanchier alnifolia	
				Larix occidentalis	
				Pinus contorta	
				Pseudotsuga menziesii	
				Xerophyllum tenax	
Lolo - 6	47.61346	-115.16873	Montana	*Salix* sp.	Geral McDonald et al.
Lolo - 7	47.40159	-115.14411	Montana	*Abies grandis*	Geral McDonald et al.
				Amelanchier alnifolia	
				Picea engelmannii	
Kootenai - 3	48.0645	-115.89574	Montana	*Holodiscus discolor*	Geral McDonald et al.
				Pseudotsuga menziesii	
				soil	
Kootenai - 4	48.56643	-114.79008	Montana	*Alnus* sp.	Geral McDonald et al.
				Abies grandis	
				Picea engelmannii	
				Pinus contorta	
				Pseudotsuga menziesii	
Kootenai - 6b	48.26979	-114.86638	Montana	*Salix* sp.	Geral McDonald et al.
Kootenai - 7	48.9499	-115.37767	Montana	*Alnus* sp.	Geral McDonald et al.
				Lonicera utahensis	
				Picea engelmannii	
				Pseudotsuga menziesii	
				Sorbus sp.	
Kootenai - 8	48.27549	-114.8446	Montana	*Alnus* sp.	Geral McDonald et al.
Flathead - 2	48.29473	-114.0207	Montana	*Acer glabrum*	Geral McDonald et al.
Flathead - 3	47.91962	-113.48981	Montana	*Betula papyrifera*	Geral McDonald et al.
Flathead - 4	47.89342	-113.71128	Montana	*Abies lasiocarpa*	Geral McDonald et al.
				Acer glabrum	
				Alnus sp.	
				Larix occidentalis	
				Pseudotsuga menziesii	
Flathead - 5	47.43331	-113.57805	Montana	*Amelanchier alnifolia*	Geral McDonald et al.
Flathead - 6	48.0188	-113.62325	Montana	*Abies lasiocarpa*	Geral McDonald et al.
				Alnus sp.	
				Larix occidentalis	
				Picea engelmannii	
				Xerophyllum tenax	
Flathead - 7	48.4274	-114.81378	Montana	*Abies grandis*	Geral McDonald et al.
				Larix occidentalis	
				Picea engelmannii	
				soil	

Bitterroot - 1	46.66	-113.85	Montana	soil	Geral McDonald et al.
Bitterroot - 2	46.19919	-113.85256	Montana	*Alnus* sp.	Geral McDonald et al.
				Amelanchier alnifolia	
				soil	
Bitterroot Ð 3a	45.56039	-114.18839	Montana	*Abies lasiocarpa*	Geral McDonald et al.
				Amelanchier alnifolia	
				Pinus contorta	
Clearwater - 2	46.71736	-115.08467	Idaho	*Pinus monticola*	Geral McDonald et al.
Clearwater - 4	46.4744	-114.97837	Idaho	*Abies grandis*	Geral McDonald et al.
				Acer glabrum	
				Holodiscus discolor	
				Prunus emarginata	
				Pseudotsuga menziesii	
				soil	
Clearwater - 7	46.39437	-115.59563	Idaho	*Abies grandis*	Geral McDonald et al.
				Acer glabrum	
				Menziesia ferruginea	
				Picea engelmannii	
				Pseudotsuga menziesii	
				Taxus brevifolia	
				soil	
Clearwater - 8	46.65686	-115.3763	Idaho	*Pinus contorta*	Geral McDonald et al.
P1	44.45123	-121.70123	Oregon	*Abies concolor/Abies grandis*	Arron Smith et al.
P3	44.42195	-121.90587	Oregon	*Abies concolor/Abies grandis*	Arron Smith et al.
				Tsuga mertensiana	
P8	42.28895	-122.08923	Oregon	*Abies* sp.	Arron Smith et al.
P9	42.324	-122.19548	Oregon	*Abies concolor/Abies grandis*	Arron Smith et al.
P10	42.4394	-122.1829	Oregon	*Abies concolor/Abies grandis*	Arron Smith et al.
				Pinus ponderosa	
				Pseudotsuga menziesii	
P11	42.52818	-122.16928	Oregon	*Abies* sp.	Arron Smith et al.
				Tsuga mertensiana	
P14	42.64923	-122.12595	Oregon	*Abies magnifica var. shastensis*	Arron Smith et al.
P15	42.71558	-122.08303	Oregon	*Abies concolor/Abies grandis*	Arron Smith et al.
				Pinus ponderosa	
				Ribes sp.	
P18	42.3786	-121.67065	Oregon	*Abies concolor/Abies grandis*	Arron Smith et al.
P22	42.60447	-121.55903	Oregon	*Abies concolor/Abies grandis*	Arron Smith et al.
				Pinus lambertiana	
				Purshia tridentata	
P28	42.67978	-121.40247	Oregon	*Pinus ponderosa*	Arron Smith et al.
P37	42.80003	-121.92159	Oregon	*Abies* sp.	Arron Smith et al.
P39	43.06332	-121.58838	Oregon	*Pinus ponderosa*	Arron Smith et al.
P45	43.17817	-121.96593	Oregon	*Abies magnifica var. shastensis*	Arron Smith et al.
				Pinus contorta	
P47	43.30132	-121.48928	Oregon	*Abies concolor/Abies grandis*	Arron Smith et al.
				Castanopsis chrysophylla	

P52	43.45297	-121.97802	Oregon	*Abies concolor/Abies grandis*	Arron Smith et al.
				Tsuga mertensiana	
P61	43.7277	-121.60357	Oregon	*Arctostaphylos patula*	Arron Smith et al.
				Castanopsis chrysophylla	
				Ceanothus velutinus	
				Pinus lambertiana	
				Pinus ponderosa	
P62	43.39163	-121.48583	Oregon	*Pinus ponderosa*	Arron Smith et al.
P67	43.6867	-120.99532	Oregon	*Arctostaphylos patula*	Arron Smith et al.
P75	43.68498	-121.33068	Oregon	*Abies concolor/Abies grandis*	Arron Smith et al.
				Pinus ponderosa	
P105	44.35388	-121.73672	Oregon	*Abies concolor/Abies grandis*	Arron Smith et al.
				Amelanchier alnifolia	
				Picea engelmannii	
				Pseudotsuga menziesii	
WO006F (JB6F)	42.42234	-122.38093	Oregon	*Abies* sp.	Josh Bronson
WO030R (Elk Creek 1R)	43.65617	-122.21479	Oregon	rotten snag	John Hanna
WO059R (Tillimook 1R)	45.62451	-123.37464	Oregon	*Alnus* sp.	John Hanna
WO077R (SOR#6)	42.77053	-122.48888	Oregon	*Pseudotsuga menziesii*	Mee-Sook Kim and Josh Bronson
WO079R (SOR#9)	42.84441	-122.48045	Oregon	*Pseudotsuga menziesii*	Mee-Sook Kim and Josh Bronson
Bovill Cemetary	46.85	-116.41083	Idaho	*Abies grandis*	Terry Shaw et al.
				Acer glabrum	
				Amelanchier alnifolia	
				Holodiscus discolor	
				Pseudotsuga menziesii	
Dick's Creek	46.60778	-116.33528	Idaho	*Abies grandis*	Terry Shaw et al.
				Pseudotsuga menziesii	
				Salix scouleriana	
Grasshopper	46.45	-115.84805	Idaho	*Abies grandis*	Terry Shaw et al.
				Pseudotsuga menziesii	
Huckleberry	48.2251	-117.93155	Washington	*Abies grandis*	Terry Shaw et al.
Kennally	44.69006	-115.98618	Idaho	*Pinus ponderosa*	Terry Shaw et al.
Newport	48.26139	-117.16111	Washington	*Pinus contorta*	Terry Shaw et al.
				Pinus ponderosa	
Pivash Creek	46.86083	-116.25833	Idaho	*Abies grandis*	Terry Shaw et al.
				Prunus emarginata	
				Pseudotsuga menziesii	
Scofield Divide	46.68278	-115.74667	Idaho	*Pseudotsuga menziesii*	Terry Shaw et al.
Skookum Lake	48.40111	-117.18945	Washington	*Pseudotsuga menziesii*	Terry Shaw et al.
Soldier Creek	47.20218	-116.45376	Idaho	*Abies grandis*	Terry Shaw et al.
				Pseudotsuga menziesii	
				Tsuga heterophylla	
Sportsman Access	47.22695	-116.51777	Idaho	*Abies grandis*	Terry Shaw et al.
Stanton	46.91441	-115.91656	Idaho	*Abies grandis*	Terry Shaw et al.
				Acer glabrum	
				Pseudotsuga menziesii	
Upper Pataha	46.24111	-117.57611	Washington	*Abies grandis*	Terry Shaw et al.

				Pinus ponderosa	
				Ribes cereum	
Vay	48.11913	-116.82207	Idaho	*Pinus contorta*	Terry Shaw et al.
Whiskey Butte	46.59139	-115.94695	Idaho	*Abies grandis*	Terry Shaw et al.
				Amelanchier alnifolia	
				Holodiscus discolor	
				Pseudotsuga menziesii	
Boise Cascade DC-2	48.13196	-117.96123	Washington	*Abies grandis*	Mee-Sook Kim et al.
Boise Cascade WC-1	48.08338	-118.00094	Washington	*Larix occidentalis*	Mee-Sook Kim et al.
Boise Cascade WT-1	48.099	-117.99414	Washington	*Tsuga heterophylla*	Mee-Sook Kim et al.
WW029R (Falls View 2R)	47.79067	-122.92543	Washington	*Pseudotsuga menziesii*	John Hanna
WW099R (Canyon Creek 1R)	47.96597	-123.20031	Washington	*Alnus* sp.	John Hanna
Abbott Creek - NA99	42.9261	-122.53314	Oregon	*Abies grandis*	Geral McDonald et al.
N.Fork Nooksack (NA236)	48.909	-121.74624	Washington	*Abies magnifica*	Geral McDonald et al.
Ochoco Divide (NA87)	44.47977	-120.34733	Oregon	*Abies grandis*	Geral McDonald et al.
Pringle Falls (NA130)	43.7043	-121.61574	Oregon	*Abies grandis*	Geral McDonald et al.
Steamboat Mountain (NA149)	46.13315	-121.7261	Washington	*Abies magnifica*	Geral McDonald et al.
T.T. Munger (NA135)	45.81448	-121.96985	Washington	*Acer circinatum*	Geral McDonald et al.
D85	47.73524	-116.51237	Idaho	soil	Geral McDonald et al.
Ida Creek	48.36319	-116.82436	Idaho	*Abies grandis*	Geral McDonald et al.
				Pinus monticola	
Pete's Creek	46.80236	-116.16285	Idaho	*Amelanchier alnifolia*	Geral McDonald et al.
				Larix occidentalis	
				Pinus monticola	
				Pseudotsuga menziesii	
				Thuja plicata	
				Vaccinium globulare	
Vasser Meadows	46.84651	-116.54166	Idaho	*Abies grandis*	Geral McDonald et al.
				detritus	
				soil	
Wet Meadows	46.86356	-116.52895	Idaho	*Abies grandis*	Geral McDonald et al.
				Menziesia ferruginea	
				soil	
Jarbidge River	41.80245	-115.40255	Nevada	*Abies lasiocarpa*	John Hanna and Patrick Bennett
				Pinus albicaulis	
				Polpulus tremuloides	
King's Canyon (1R)	39.12504	-119.88329	Nevada	*Pinus jeffreyi*	John Hanna and Sara Ashiglar
Boulder Creek ID (1F)	45.14788	-116.4006	Idaho	*Pseudotsuga menziesii*	John Hanna
Boulder Creek ID (2F)	45.10132	-116.44014	Idaho	*Pseudotsuga menziesii*	John Hanna
Cold Springs Saddle (2R)	44.5666	-116.21201	Idaho	*Populus trichocarpa*	John Hanna
Trail Creek (1R)	45.14895	-116.34777	Idaho	*Abies grandis*	John Hanna
E. Branch Weiser River (1R)	45.05525	-116.43622	Idaho	*Abies grandis*	John Hanna
Hard Creek (1R)	45.15756	-116.16821	Idaho	*Populus trichocarpa*	John Hanna
Last Chance (2R)	44.99791	-116.19721	Idaho	*Pseudotsuga menziesii*	John Hanna
Little Goose Creek (1R)	44.95815	-116.17284	Idaho	*Abies grandis*	John Hanna
Mica Creek (1R)	44.61055	-116.23905	Idaho	*Abies grandis*	John Hanna
Outlet Creek (1R)	45.09509	-116.02855	Idaho	*Abies lasiocarpa*	John Hanna

Pearl Creek (1F)	45.0894	-116.04496	Idaho	*Abies lasiocarpa*	John Hanna
Pine Creek ID (1R)	43.77963	-115.79341	Idaho	*Populus trichocarpa*	John Hanna
No Business (2R and 2F)	44.7496	-116.18869	Idaho	*Abies grandis*	John Hanna
No Business (3F)	44.74962	-116.18867	Idaho	*Abies grandis*	John Hanna
Sand Pit (1R)	44.99841	-116.06029	Idaho	*Picea engelmannii*	John Hanna
Shovel Creek 1F	41.81837	-122.19527	California	*Abies concolor*	John Hanna and Patrick Bennett
Rat Trap Gap 1R	40.20723	-122.94346	California	*Pseudotsuga menziesii*	John Hanna and Patrick Bennett
Northgate 1R	41.47206	-122.18864	California	rotten stump	John Hanna and Patrick Bennett
McMullen 1R	40.64297	-121.69852	California	*Abies concolor*	John Hanna and Patrick Bennett
Jack's Backbone 1R	40.66402	-121.66862	California	*Abies concolor*	John Hanna and Patrick Bennett
